# Taking mHealth Forward: Examining the Core Characteristics

**DOI:** 10.2196/mhealth.5659

**Published:** 2016-08-10

**Authors:** Teaniese Latham Davis, Ralph DiClemente, Michael Prietula

**Affiliations:** ^1^ Public Health Sciences Institute Morehouse College Atlanta, GA United States; ^2^ Department of Behavioral Sciences and Health Education Rollins School of Public Health Emory University Atlanta, GA United States; ^3^ Goizueta Business School Health Initiative Emory University Atlanta, GA United States

**Keywords:** mobile health, eHealth, mHealth, health policy, health technology, text messaging, public health informatics, telehealth

## Abstract

The emergence of mobile health (mHealth) offers unique and varied opportunities to address some of the most difficult problems of health. Some of the most promising and active efforts of mHealth involve the engagement of mobile phone technology. As this technology has spread and as this technology is still evolving, we begin a conversation about the core characteristics of mHealth relevant to any mobile phone platform. We assert that the relevance of these characteristics to mHealth will endure as the technology advances, so an understanding of these characteristics is essential to the design, implementation, and adoption of mHealth-based solutions. The core characteristics we discuss are (1) the penetration or adoption into populations, (2) the availability and form of apps, (3) the availability and form of wireless broadband access to the Internet, and (4) the tethering of the device to individuals. These collectively act to both enable and constrain the provision of population health in general, as well as personalized and precision individual health in particular.

## Introduction

### Background

The issues, problems, and opportunities in health care are composed of many interacting components such as technologies, economics, players, business models, practices, policies, and laws. These components are constantly shifting and influencing one another. A rapidly emerging, and often essential, part of health care involves the growth of information and communications technologies (ICTs) [1,2]. Electronic health, or eHealth, encompasses a remarkably wide range of interpretations related to health care [3,4] and ICTs [5,6], with one study documenting over 50 uniquely different definitions for the term “eHealth,” such as the application of e-commerce to health care and pharmaceuticals, the use of new media technologies for social policy, and the integration of the Internet into health care [7]. Such diverse interpretations can lead to multiple operational definitions of eHealth, negatively influencing generalizability of study results [8]. This is complicated by alternative terms appearing in the literature and in practice, with those terms also evolving as the technology changes. For example, the United States federal government and other agencies use the terms “telemedicine” and “telehealth” to generally refer to applications of ICTs in the practice of medicine, but are attempting to reach consensus on the use of the terms [9]. In one bibliographic analysis, the term “telemedicine” was used more than “eHealth” in English-speaking countries, whereas “eHealth” was more popular in non–English-speaking countries; “eHealth” is trending at a higher rate overall [10,11].

Therefore, any analysis or review of telemedicine, telehealth, or eHealth programs must be cautious when comparing outcomes, as there are interpretations supported by the definitions, the types and implementations of the technology, and how it was used in the particular health intervention context. These should not be treated as exogenous factors. Within this terminology health care, individual, clinical, and public health are often confounded, thus covering a wide range of health application contexts including financial and business sectors [12].

Regardless of the broader terms (eHealth, telemedicine, telehealth), one term has emerged and tends to refer to a specific class of technologies and the associated ways in which the class is used with respect to health—mobile smart phone telephony or mHealth. In this paper, we briefly examine the etymology of the term “mHealth,” followed by an examination of technologies used in mHealth. Then, we describe core characteristics of mHealth’s current technologies in terms of their potential relevance for the general “health of the public” which has implications for health care at large, to the localities of health practice, and to individualization of information, clinical care, and behavioral intervention. We present this paper as augmenting other discussions regarding strategies taking mHealth forward [13,14]. As mHealth evolves in methods for engaging in prevention, care, treatment, and monitoring of health, these characteristics will help guide development toward more effective and efficient health solutions. As ICTs evolve in their forms, capacity, and presence, these characteristics will help health professionals instantiate components of their health solutions to realize their health goals. In short, this paper addresses the following question: What are the core characteristics of mHealth and why are they important?

### Etymology of mHealth

mHealth typically includes applications in both public health and clinical medicine, so the distinction can be blurred. In clinical medicine, mHealth includes health costs, health delivery, Health Insurance Portability and Accountability Act (HIPAA) or health information technology, and health telematics [15,16]. Although no standard definition of mHealth exits, both the World Health Organization and National Institutes of Health have offered reasonable and overlapping definitions of mHealth consistent with the view in this paper. Table 1 includes additional agency definitions. mHealth generally involves the use of mobile phones which, depending on the particular phone, directly support audio, video, photography, geolocation, sensors (proximity infrared, touch, accelerometer, ambient light illuminance, humidity, three-axis gyroscope, temperature, magnetometer, geomagnetic, ultraviolet exposure, light spectral, gestural-infrared, barometer, pedometer, pulsometer, heart rate, radiation detection), Internet or Web access, and various forms of texting, with locally running mostly third-party applications, or “apps,” as the emerging terminology standard [17].

Ancillary devices can be connected to mobile phones, including “watches and bands,” and communicate with the apps to provide unique and often innovative capabilities, such as blood pressure monitors, pulse oximeters, blood glucose meters, environmental exposure measures (for asthmatics), microscope for remote diagnoses, single lead electrocardiogram (ECG), sleep monitor, and an ingestible biomedical sensor [18], or integration with existing devices that can communicate with apps such as Google and Novartis’s “smart” contact lens that also monitors blood sugar [19,20]. Also consider other types of nonmedical devices that are attached to or communicate with a mobile phone sold as add-ons with or without medical claims, possessing medical device capability, such as thermal imaging [21] or arrays of integrated, wearable sensors capable of streaming data in real-time to the mobile phone app [22]. New telemedicine or telehealth mHealth apps are emerging as a consequence of policy (eg, the Patient Protection and Affordable Care Act, economic returns or displacements, regulatory structures), business opportunities, and technological innovations [23-26]. This is especially prominent with devices adept at exploiting the capabilities of mobile phones [27].

**Table 1:** Definitions of mHealth (mobile health).

Agency example	Definition
Word Health Organization	mHealth involves the use of and capitalization on a mobile phone’s core utility of voice and short messaging service (SMS) as well as more complex functionalities and apps including general packet radio service (GPRS), third and fourth generation mobile telecommunications (3G and 4G systems), global positioning system (GPS), and Bluetooth technology [28].
US National Insitutes of Health	At NIH, we think about this really as diverse application of wireless and mobile technologies designed to improve health research, health care services, and health outcomes, and I think this is really important because it is not just mobile phones. You can think of it as sensors, any kind of sensors you can think of [29].
US Federal Communications Commission	The use of mobile networks and devices in supporting e-care. Emphasizes leveraging health-focused applications on general-purpose tools such as mobile phones and SMS to drive active health participation by consumers and clinicians [30].
US Department of Health and Human Services	The use of wireless technologies, such as mobile phones, personal digital assistants, and netbooks, for improving health [31].
mHealth Working Group	mHealth is the use of mobile technologies in public health and health service settings [32].
European Coordination Committee of the Radiological, Electromedical and Healthcare IT Industry (COCIR)	COCIR regards mHealth as a subset of eHealth and defines it as the provision of eHealth services and information that relies on mobile and wireless technologies [33].
Adopted by mHealth Regulatory Coalition	Programs (apps) that deliver health-related services using mobile phones and tablets. Some apps offer advice and tracking functionality for healthy living. Some are designed to transmit information between doctors and patients (eg, glucose readings for diabetes management) [34].

Advancements in the mHealth infrastructure are bolstered by support of the underlying operating system (OS) technology in mobile phones. In September 2014, Apple Health’s HealthKit platform released with the iOS 8 systems development kit (SDK) offered a set of integrated software to collect specific types of health data, allowing developers to create iPhone apps that directly interact with the OS’s health-related, built-in resources to securely acquire, retain, and transmit health data. After a rocky start [35,36,37], Apple strategically collaborated with the Mayo Clinic [38], Nike [39], and Ochsner Health System [40]. The latter was significant as it also included partnering with Epic Systems to integrate their electronic health record (EHR) with HealthKit, a leading EHR vendor handling approximately 53% Americans’ EHRs [41]. Other leading health centers, such as Stanford and Duke, quickly followed by launching clinical trials using HealthKit, 14 hospitals piloting HealthKit for institutional data integration, and at least 600 HealthKit-based apps were developed [42,43]. Apple and IBM announced a partnership to build enterprise mobility apps, bringing IBM’s experience in data analytics and health care to Apple’s mobile iPhone and iPad platforms [44,45]. With the introduction of iOS 9, the iPhone provided wider support for women’s health issues [46,47] and iOS 10 supports Health Level 7 (HL7) standard for Continuity of Care Documents, which is the EHR standard for data exchange [48].

In March 2015, Apple released ResearchKit, an open source software framework that facilitates app development in the context of medical research studies [49]. ResearchKit has an integrated informed consent module, capturing a participant’s signature, and a customizable user interface, allowing data capture such as tacit data from sensors, from user-engaged active tasks, and from surveys, then uploads data to a server for retention and data analyses. By all accounts, ResearchKit’s focus on clinical research and researchers has been quite successful, as it addressed several difficulties that HealthKit encountered with this target group. For example, ResearchKit facilitates the “remote” acquisition of participants, their consent, and their data for clinical trials [50,51]. Recently, ResearchKit has been updated to now accommodate genetic data from 23andMe [52].

Finally, in March 2016, Apple has introduced CareKit, a framework for building apps that “enable people to actively manage their own medical conditions” especially patients and their caregivers, including core modules to track their health-related events (eg, taking medication, conducing physical therapy) and track their own feelings and symptoms (eg, surveys, photos, motion quantification, temperature) [53]. Unlike HealthKit, which addresses basic health tracking (eg, exercise, food intake, weight), or ResearchKit, which is more a “connection to participants and their data” type of research facilitator processes, CareKit has a patient-centered focus, offering an Insight Dashboard that maps symptoms into action items, and connectivity to physicians, care teams, and family members targeting chronic conditions, such as home monitoring of Parkinson’s disease, and postsurgical care [53].

Samsung’s Android S Health activity tracking app, once available only on Samsung mobile phones, is now available in the online Play Store for all Android phones, though functionality is dependent on available sensors. S Health offers its own Digital Health SDK [54], with more of a nuance toward fitness and health, like Google Fit [55]. Separately, Samsung has partnered with the University of California, San Francisco, and imec (a leading biosensing research institute) for the Samsung Digital Health Initiative, which includes a new open reference design platform for health (Samsung Architecture Multimodal Interactions) as a data broker across apps, and a wearable technology reference design, Simband, based on its Gear watch design [56]. Simband is hardware designed from the ground up as a true digital health device. However, it is not yet a directly commercial product, but is a flexible reference device, based on the Linux-based Tizen standards–based software platform, for different types and collections of sensors, such as ECG, galvanic skin response, bio-impedence, photoplethysmogram, and other or new types generating unique data streams that can be integrated (and analyzed) across multiple devices by third party developers [57].

Finally, Google has entered the market with an initial version of the Google Fit SDK with the release of Android 5.0 Lollipop, offering developers support for sensor data acquisition (phone-based and attached wearables), cloud-synced data collection-backup, and history presentation-tracking [58], but likely to be integrated with Android Wear devices [59]. Google is also partnering with the Mayo Clinic on constructing and reviewing medical information in their Knowledge Graph system for answering health-related queries on the Web using “rich content” data [60,61]. This is consistent with Google’s mission statement, “to organize the world's information and make it universally accessible and useful.” It also reflects the Pew Research findings that 72% of US Internet users look on the Web for health information, 77% of those start with a generic Internet search using a search engine, and 35% of US adults have attempted diagnosis from Web-based information [62]. Furthermore, the number of search queries made on mobile devices has overtaken desktop search queries [63]. In parallel, Google’s parent company, Alphabet, formed Verily Life Sciences (formerly Google Life Sciences) for biomedical research, is developing wearable technologies beyond fitness trackers that directly address data needs of medical professionals and evidences a shift to a disease-centric focus, such as their “capicola” wrist-mounted health tracker, partnering with Novartis to develop glucose monitoring contact lenses, to treat and manage diabetes [64-66].

The implications for mHealth for these types of integrated health platforms are only beginning to be realized, but Apple has taken the lead in hospital pilot programs, many of which involve close (but remotely) tracking and management of individuals’ conditions [42,52]. Two major factors motivate the growth of mHealth apps. First, the overall general movement of health prevention likely drives demand, which is fueled by a combination of individual concerns for health as well as economic incentives, such as health care reform reimbursement mechanisms for physicians and support for wellness programs. There is increased value in acquiring personal activity and health data for individual awareness, growth of wellness programs in the workplace, and physician utility in individualization of health care advising, especially for costly chronic diseases [67]. Second, health care organizations are concerned about operational inefficiencies and outcome improvement. Data acquisition, movement, integration with EHRs, and cloud-based analytics within health care organizations are a critical part of operational and outcome-based concerns, leading to a projection that 65% of consumer transactions with health care organizations and practitioners will be mobile by 2018 [68].

As the health technology landscape is rapidly, constantly, and unpredictably changing, we suggest that successful mHealth engagements can benefit by (1) focusing on the core characteristics of mobile phone technology and (2) understanding how these characteristics are successfully engaged and integrated. Mobile phone technology is often essential for improving the effectiveness and efficiency of achieving health goals for individuals, groups, states, or nations.

### Core Characteristics of mHealth

#### Overview

We suggest that the leading core characteristics of mHealth are the following: (1) penetration into populations, (2) availability of apps, (3) wireless broadband access to the Internet, and (4) tethered to individuals. The core characteristics are summarized in Table 2. Many capabilities are built upon these characteristics such as social network communication structures, data acquisition mechanisms, and varieties of functional apps. Furthermore, these capabilities exist in “layers” where capabilities are built on other capabilities (eg, social networking functionality built on collective use of specific apps and Internet access tethered to individuals, health apps are built on the underlying functionality of the OS and hardware) or “assemblies” where functionality is based on devices attached physically (hardware) or electronically (software). Not all characteristics are equally available to all populations at equal levels of service. State-level statistics often do not reflect the heterogeneity of technological forms and access within its borders. However, mobile telephony with Internet access is the most rapidly developing and spreading form of telecommunication-computational technology. It is a small and increasingly powerful computer with a radio attached that you can fit into your pocket. Given the correct infrastructure, it can communicate rapidly with individuals and things around the world.

**Table 2:** mHealth core characteristics for health care.

Representative topics	Health care implications
**Penetration into populations**	Unprecedented communication access to population and subgroups, possible differential access by subgroups, differential mobile phone capability or dependence by subgroups
**Availability of apps**	General purpose computational capabilities of increasing sophistication of functionality and data acquisition, reporting, local analysis; ease of access to apps; increasing number of health-related devices connected to mobile phones
**Wireless broadband access to the Internet**	Access to full Internet resources; increasing sophistication, amount, and speed of communication in general, and data communication in particular; external device connectivity; Internet of things (devices) capable of direct Internet communications
**Tethered to individuals**	Decreasing delays in communication with specific individuals; tailoring to, and data captured by and about, individuals; location, physiological and psychology states, behavioral, and context awareness

### Core Characteristic 1—Penetration Into Populations

The first core characteristic of mobile phone technology is its *penetration into populations* in the United States and around the world, affording substantially higher basic connectivity and connectivity quality (eg, reach, continuity of service, reduced connectivity delays). Wireless penetration in the United States is estimated at 110 mobile phone subscriptions per 100 population [69] and estimates place the ownership of mobile phones by American adults approaching 80% [70,71]. In 2015, the Center for Disease Control and Prevention’s National Health Interview Survey reported over 47% of American homes were wireless only (meaning they had neither cabled phone nor cabled Internet access), where 55.3% of homes with children are wireless only [72]. Note that such figures vary across reporting agencies depending on the usual factors, such as timing, sampling, operational definitions, and methodological differences.

Globally, the International Telecommunications Union (ITU) estimates that the mobile phone penetration rate is 96.8% for the world population, 120.6% for developed countries, and developing countries at 91.8%. The number of world mobile phone subscriptions (ie, the number of SIM cards used in each country) is roughly equivalent to the world population [73] and the number of subscriptions exceed the total population in several counties in Central and Eastern Europe, Western Europe, Latin American, Middle East, North American, and Asia Pacific, excluding India and China [74]. Both penetration and type of access vary widely. For example, although Africa has an average penetration rate of 69%, sub-Saharan Africa’s sovereign states range from 40% to 96% in households that have at least one mobile phone and represent the second largest mobile technology market after Asia [75]. In South Africa and Nigeria, mobile phone ownership by adults is almost equivalent to ownership in the United States, but smartphone ownership in sub-Saharan Africa lags behind from a high of 34% in South Africa to a low of 8% in Tanzania and 5% in Uganda [76]. Similar situations exist in South America, where mobile phone penetration is generally high, but smartphone penetration lags, and sometimes substantially. For example, consider the 2015 estimated rate for mobile phone penetration (% of population with device) versus smartphone penetration (% of population who have mobile phones) in the following countries, respectively, is: in Chile (73.3%, 55.5%), Argentina (70.6%, 43.5%), Columbia (69.3%, 51.4%), Brazil (69.2%, 35.8%), Mexico (67.2%, 47.4%), and Peru (62.6%, 33.5%) [77,78].

Regarding health, changing preferences and expectations play a large part in adoption of mHealth services, as well as sub populations’ differential access to smartphone technology and its infrastructure. Knowledge of likely access can be important. For example, an estimated 88% of American teens between the ages of 13 and 17 years have access to mobile phones and 73% own a smartphone [79], but this age group has different behavioral risk patterns than older populations regarding human immunodeficiency virus infection behavior. Such differences impact intervention design approaches and what can be supported by the target population and technology platform [80]. The number of “Millennials” (ie, ages 18 to 34 years) in the United States will increase to over 75 million and outnumber every other generational category in 2015 [81]. The *2015 State of the Connected Patient* [82], reported that 40% of Millennials did not believe their primary care physician “would recognize them if they passed each other on the street” and 60% of Millennials preferred a video chat with their physician in place of an in-office visit. Among US teens aged 13-18 years, the Internet is the primary source of health information with 84% obtaining health information from the Internet and one in three teens have changed their behavior based on that Web-based health information [83]. Such information search capability requires Internet access (Core Characteristic 3).

Mobile connectivity can be much more than a social convenience. Consider Africa, which has the lowest Network Readiness Index as defined by the World Economic Forum [84]. The growth in basic mobile-based access affords simple messaging, which reduces the need for face-to-face medical visits, thus reducing health care costs due to reduced transportation needs and reduced diagnostic or referral delays for controlling Malaria outbreaks, especially in rural areas [85,86]. In Africa, South America, and Asia, there is a disproportionate number of poor people who lack direct access to the financial infrastructure—the “unbanked.” Consequently, mobile access gives access to information and services, such as sending and receiving payments, allowing them to directly participate in, and gain from, a developing economy [76,84]. Relatedly, it is estimated that “75% of the world’s poor rely on agriculture for all or some of their household income,” so even simple (SMS-based) text messaging allows informational exchange with specialists to increase agricultural productivity and, consequently, income [87].

Although the costs of owning smartphone technology are decreasing, and, perhaps as a consequence, ownership of mobile phones is increasing, the penetration and adoption of this technology is not uniform. It is essential to know the adoption patterns of the target population for the technological platform itself.

### Core Characteristic 2—Availability of Apps

The second characteristic of smartphone technology is the *availability of apps* installable on the device. At its heart, we noted a “smartphone” is basically a computer with radio communications capabilities. Thus, smartphones have the functions and capabilities of a general purpose computer platform, enabling third-party developers to build and run their own programs (“apps”) exploiting the functionality of the particular device. Apps must be built specifically for the device’s OS (ie, Apple iOS, Android, Windows, Blackberry, Symbian).

The software driving smartphones is the OS. Note that iOS is unique to Apple, Blackberry OS is unique to Blackberry, but several phone manufacturers use Android OS (eg, Google, Samsung, HTC, LG, Motorola, and now Nokia) and Microsoft Windows Phone OS (eg, Samsung, Nokia, HTC, Huawei). Symbian OS and its primary supporter Nokia dominated the mobile phone market from early 2000s to 2010 though it is now on a substantial downward trend [88]. Given the Android open source software availability [89] and Android “remerging” into the Linux open source project [90], other Linux-Android OS phones are emerging with uniquely branded OSs, such as the Ubuntu phone [91]. As noted, Samsung is also developing phones and devices that use the open source Linux Tizen OS [92].

Furthermore, apps can be constructed to interact (acquire data and drive functionality) with connected devices, wired or wireless. As smartphones comprise a more expensive, but more capable, subset of the general mobile phone devices, the penetration levels are comparatively lower than smartphones, but as we noted earlier they are rapidly increasing due to relative price declines. In December 2015, US estimates indicated smartphones were owned by 79.3% of mobile subscribers, with Android phones holding 53.3% of the market, Apple iOS with 42.9%, Microsoft with 2.9%, and BlackBerry with 0.9% [93]. Worldwide second quarter estimates place Android comfortably in the lead with 82.8% of smartphone OS, Apple with 13.9%, Windows with 2.6%, and BlackBerry with 0.3% [94].

There are a remarkably large and growing number of general apps available under the 2 dominant platforms—Apple (1.5 million) and Android (1.6 million)—as of July, 2015 [95]. Although the market share for the Windows phone is dominated by Android and iOS, the use of apps may be more fluid as Microsoft is targeting the development of apps for both Android and iOS devices [96] as well as providing technological support (via software development kits) to port iOS and Android apps to Windows [97].

From a health care perspective, smartphone apps reside within increasingly powerful (mobile, hand-held) hardware-software environments, allowing sophisticated data acquisition, communication, and computation-intensive processes to be done locally, such as various types of numerical and non-numerical calculations, data analytics, display graphics, and video, along with megabytes to gigabytes of local data storage. For example, the growth of mobile phone capabilities can bring important analytics to drive treatment decisions in outbreaks in rural or underserved populations, such as the use of a microscope app that analyzes videos of whole blood samples for microfilarial parasite *L loa* motion, quantifying density for determination of treatment within 2 minutes [98]. Similar mobile phone apps are in design, development, or production for in vitro and environmental testing directly (or indirectly with attached ancillary devices) supporting onsite detection of pathogens, such as disease markers via nucleic acid isolation, gold nanoprobe Tuberculosis diagnostics, microchip ELISA detection of ovarian cancer via HE4 biomarker and other cancer cell diagnostics, fluorescent imaging cytometry, lateral flow immunochromatographic assays, loop-mediated isothermal amplification genetic testing, and acoustic wave immunoassay to name a few [99]. Thus, even in resource-poor environments, mobile phone apps in the hands of health care workers can bring critically needed point of care or point of need analytics previously unavailable to underserved populations.

The nature of this reach is enhanced by the sophistication of the apps that can be run on the mobile devices given their computational capabilities, and these computational capabilities are escalating. Estimates suggest that the Apple iPhone 6’s A8 chip is 50x faster than the chip in the original iPhone and its graphics processing unit is 84x faster [100], but the Apple iPhone 6s’s A9 SoC (System on Chip) has an estimated 50% increase in processing performance and a 90% increase in graphics performance over the A8 [101]. The ARM Mali-T760 MP8 graphics processing unit in the Samsung Galaxy S6 can generate a peak performance of 302 Gflops [102], roughly equivalent to the supercomputers in 1996 [103], whereas IBM’s Deep Blue supercomputer, which bettered the world chess champion Gary Kasparov in 1997, has an estimated performance of 11.4 Gflops and was the 259th fastest supercomputer in 1997 [104], emphasizing how the technological capability is used, the critical role of programming.

For these devices, ancillary storage and speed of types of secure digital (SD) cards, the typical detachable storage medium, is also expanding along with the transfer speed. The size of the card is contracting, with the cost per storage unit decreasing as the next wave of improvements enter the market. In 2003, SD had a capacity of 512 megabytes, but disk capacity has increased 1000-fold, where SanDisk’s 512 gigabyte SD cards are temperature tolerant (−13° to 185°F), waterproof, shockproof, and x-rayproof [105]. This capacity means the card approximates the size of a postage stamp, any given mobile phone could have direct access to over 458 million pages of text (1200 characters per page) or a complete copy (snapshot) of all Wikipedia including images, for offline access [106] with space left over for either 128,000 average size ePub formatted books (3 Mbytes) or over 4500 five-minute YouTube videos at 720p. Thus, the information “at your fingertips” can be rather large. However, SD technology is quickly approaching one of the popular SD standards (SDHX) limit of 2 terabytes (2048 gigabytes).

The arrays of different sensors and associated computational analysis contained within the mobile hardware or attached to the phone (directly or wirelessly) complement the raw computing power and data storage capacity. The ability to gain access to mobile broadband capabilities opens the floodgates of possibilities as the connectivity and local computational capabilities allow true point-of-care enhanced capabilities; communication nor computation are tethered to landlines. Mobile phones are however tethered to individuals. The possibility of personalization of public health, real-time data acquisition, and a host of other possibilities relevant to assessing, monitoring, and reacting to individualized contexts are just beginning. Issues of data privacy, HIPAA defined risk, app functionality risk and validity, and beyond are rapidly rising to the top of development agendas [107]. Most of the legal barriers, because of technology available when they were written, are oriented toward the electronic protection and privacy of patient information held in health care databases. Yang and Silverman [108] warn that when software is voluntarily downloaded and installed (as an app), accessing data from that app about the user may not be considered “unauthorized” as it is sometimes wrongly assumed under the Computer Fraud and Abuse Act of 1986. In fact, they even conclude the Electronic Communications Privacy Act “probably does not protect against the access or acquisition of data by the developer of a private app” (p. 224).

Most users (and uses) do not demand high computational performance of mobile phones, but it is likely that this demand will escalate with the user-facing capabilities of the health-related apps. Consequently, types of health services that can be offered via these platforms are increasing in number and sophistication. Referring again to the *2015 State of the Connected Patient* report [82]: 63% of Millennials would be interested proactively providing their health data from Wi-Fi or wearable devices to their doctor or provider so they can monitor their well-being and 71% would be interested in a doctor giving them a mobile app to actively manage their well-being for preventative care. Such interests require demands going beyond simple local data capture and reporting (eg, “fitness” apps), but more sophisticated data acquisition and processing coupled with sufficient analytical and communication capabilities. Finally, it is worth noting that an IMS Institute reports that despite the 100,000+ of health apps, just 36 apps accounted for almost 50% of the downloads (ignoring use), and 40% of the apps had less than 5000 downloads [109].

### Core Characteristic 3—Mobile Broadband Access

The third characteristic of mobile phone technology is the ability to *access the* Internet or World Wide Web via mobile broadband. It is not sufficient that devices are portable, powerful, and capable to run the appropriate apps, but it is often the case that they must also be able to communicate via rapid Internet pathways. The technological sophistication or maturity of a region (defining its capabilities) is determined by the particular mix of its mobile technologies [110]. Note that the term “wireless broadband” should generally not be used in place of “mobile broadband” (or mobile cellular broadband) as wireless broadband can include fixed-wireless and satellite technologies, although some satellite broadband technologies can be mobile.

The ITU generally defines mobile broadband as download data speeds of at least 256 Kbit/s using the Internet protocol and access to the “greater Internet” (ie, World Wide Web) via the Hypertext Transfer Protocol, but solely having standard short messaging service (SMS) does not count as broadband as it is a mobile-based voice protocol and does not require Internet access [111]. Mobile broadband is also defined in terms of technological generations (G), where 2G, 2.5G, 2.75G (1990s) technologies were the first digital cellular networks with speeds in the ITU range just noted and represents an earlier 2011 definition yielding 90% world coverage [112]. As with any evolving technology, definitions must change as the categorical characteristics of the technology change [113,114], so it is important to understand the operating definition of mobile broadband being used, and whether the reported statistics refer to overall capacity (eg, gigabit levels attributed to 802.11ac) or actual expected delivery rates for individuals under operational conditions and distance. In particular, many definitions specify or assume at least 3G technologies [115], with Long Term Evolution and Long Term Evolution-Advanced being the current fastest widely deployed mobile broadband technologies in terms of overall capacities and the closest to becoming the global base standard (there are different deployment forms) for mobile broadband [116]. In addition, intermediate steps taking advantage of current standards have yielded niche standards such as WiGig (802.11ad), which is a very fast (7 gigabits per sec, low latency), but short range (1-7 m) designed for close connectivity, such as eliminating cables [117]. On the other hand, the forthcoming next-generation 802.11ax wireless specification is not only seeking increases in network capacity but also seeking to create larger “data pipes” to individual devices, thus radically increasing not only the network capacity but also the average data rates to individual devices [118].

When considering newer baseline definition of mobile broadband subscriptions (at least 3G networks), the ITU of the United Nations estimates that 47.2 per 100 inhabitants of the world’s population is to be subscribed in 2015, with 89% of the world urban population (4 billion) covered but only 29% of the rural population (3.4 billion) covered, but substantial distributional differences between the developed countries (86.7 per 100 inhabitants), developing countries (39.2 per 100 inhabitants), and the least developed countries (12.1 per 100 inhabitants), where Africa is the sole region where mobile broadband is below 20 per 100 inhabitants (though within-regional differences occur) [115]. However, comparisons are complex as there is still a great deal of variation in how “mobile broadband” is defined, how specific technologies are classified, and how measurements are conducted [119]. Regardless, Ericsson predicts that by 2021, 85% of all smartphone subscriptions will be for mobile broadband, but whether it plays a replacement role for fixed broadband or a complementary role depends on the particular segment [74].

It is important to understand how these characteristics map to target populations. For example, there are distinct issues with the lack of rural broadband (wired or wireless or mobile) in the United States, where 53% of rural Americans (22 million people) lack such access, as do almost two-third of US territories and Tribal lands [120]. A 2015 Pew survey found that 54% of sampled African American homes had broadband, a decline from 2013, and this percent varied by household income, ranging from a low of 41% (income < $29K) to a high of 80% ($50K ≤ income ≤ $75K) [121]. The same survey showed that almost 20% of African American households have smartphones, but no broadband.

There is an emerging trend of smartphone dependence among younger adults, nonwhites, lower income, and lower educational attainment, where these devices are the sole mechanism for both Web-based access (often at slow speeds) and phone calls (elimination of land lines) for communication, but also are less likely to be covered by health insurance or have a bank account [122]. The decline of landlines in the United States was further evidenced by a Centers for Disease Control and Prevention survey that estimated 47.1% of children and 39.1% of adults live in wireless-only households, with 56.2% of poor households and 53.1% of Hispanic households were wireless-only [72]. Two consequences of decline in landline use, sometimes overlooked, are (1) the loss of community revenue typically accrued by local telecommunication taxes, as federal law prohibits state and local taxation of Internet data, including communication apps such as Skype, FaceTime, WhatsApp, and Twitter [123] and (2) potential bias in “phone-based” surveys reliant on landline registers [72,124].

The penetration of mobile broadband worldwide is expected to surpass fixed-broadband by 2017 [125]. In fact, the 34 member countries of the Organization for Cooperation and Economic Development now report an average of 81.3% mobile broadband penetration (wireless subscriptions per inhabitant), with 7 members averaging above the 100% mark [126]. Collectively the Organization for Cooperation and Economic Development member countries’ population is approximately 1 billion people, and the results necessarily do not include data from other important regions, as Africa, India, Brazil, China, and Russia. For example, in the least developed countries, fixed-broadband remains less than 1% and escalation to mobile broadband is the sole option [115].

Regarding health care, mobile broadband is essential for accessing the Internet resources and supporting the sufficient bandwidth for communication capabilities of data-intensive mobile phone app, such as video, without the need for, or reliance on, geospecific landlines. For example, the Mobile MIM iOS app allowing viewing X-rays, ultrasounds, neuroimaging on iPads, or iPhones by medical professionals, but developers also provide VueMe iOS apps for secure patients to view and discuss images with their health providers without physical presence required [127].

Despite the increasing capabilities of mobile phones, attached devices offer another route to point-of-care accessibility via mobile technology, allowing local complex data acquisition (via the combined capability of the attached device and the mobile phone) by a health care professional. The MobiUS SP1 mobile phone app interacts with a hand-held ultrasound device, allowing local scanning and transfer of imaging via Wi-Fi, cellular or USB to PC connection [128]. Dexcom’s Apple Watch app displays patterns of glucose readings. It receives data from Dexcom’s Continuous Glucose Monitoring system that captures glucose levels by a subcutaneous sensor, up to 288 readings per day, and relaying the information (and any warnings) to up to five additional “followers” [129]. The AliveECG app (with the attached device) allows individuals to take their own ECG with their Android or iOS smartphone (or tablet), which then analyzes the data to determine if atrial fibrillation (a leading cause of stroke) is detected [130]. Proteus Digital Health has both an ingestible sensor-enabled pill that works with an external patch that detects a signal that is generated from the pill when ingested (reaches the stomach) and records rest or activity patterns (steps, heart rate) that are sent via Bluetooth to the caregiver’s, patient’s, or physician’s mobile phone app, as well as to a secure database accessible by authorized health care professionals [131]. Propeller Health’s sensor attaches to most asthma and COPD inhalers, capturing time and geolocation data of use (and other data, such as user-supplied medications), linking to an app that can share that data with health care partners or anonymously (eg, capturing timing and location data across users signaling environmental situations), but the app also provides information on adherence and education [132]. Medronic’s MiniMed system connects its small insulin pump and continuous glucose monitor to a diabetics iPhone, sending pump and sensor information to the iPhone every 5 minutes, and sending history information to their CareLink health care “partners” every 24 hours, or preset text messages immediately if glucose levels exceed tolerance levels [133]. In addition, Medtronic is also partnering with Samsung to develop Android apps for that device series [134].

All of the previously noted devices are examples that have been approved, or are being reviewed for approval, by the US Food and Drug Administration at the time of this writing, which speaks to the separate topic of how smartphone technology (directly or indirectly) takes on the role of a regulated medical device [135]. But Internet connectivity is an ever-emerging architecture. Every smartphone can act as a wireless hotspot affording a gateway for other smartphones or computers or attached devices. To complicate things, Bluetooth 4.2 allows devices to securely connect to each other and to the Internet via IPv6/6LoWPAN (IPv6 over Low-Power Wireless Personal Area Networks) via direct connection to any gateway device (eg, a router) and avoid the necessity of a smartphone entirely, thus opening the communication door for the “Internet of Things”—sensors, instruments, devices, and attachments that have the capability of interacting directly with the Internet [136].

### Core Characteristic 4—Tethered to Individuals

The final characteristic of mobile phone technology is that mobile phones are usually tethered to *individuals*, though the validity of this characteristic is dependent on the heterogeneity of the penetration of this technology in certain populations. With respect to penetration, consider that mobile phone technology differs critically from landlines on this metric because mobile phones are associated with individuals and not residences (requiring a permanent, physical location), so there is usually a 1:1 mapping between individuals and mobile phone use (but this mapping can also be 1:N, when individual users have more than one mobile phone account per SIM card). In addition, a US Gallup Panel in July 2015 revealed that on the average 11% checked their mobile phone devices every few minutes (22% of those between the ages of 18 and 29 years), and an addition average of 41% reported checking a few times an hour (51% of those between the ages of 18-29) [137]. Nevertheless, the assumption of individual tethering is likely a valid default assumption sans conflicting evidence.

At a basic level, this affords an efficient technological mechanism for 2 health purposes: data monitoring-acquisition and intervention delivery. Regarding the former, individual tethering allows “immediate” acquisition of personal event data, actively or passively. For example, various types of experience sampling or ecological momentary assessment methods have emerged to capture individuals’ self-reporting episodic descriptions (event based, time based) of behaviors and experiences in “real time” (ie, to avoid delayed, retrospective autobiographic approaches that may be biased in recall or inaccurately reported) in real-world contexts, such as diaries (paper or electronic), telephone calls, generally emerging in the 1990s [138-140]. Mobile phones are now serving as a natural platform for applying such data acquisition approaches for conditions as tobacco use [141], alcohol use [142], development of virtues [143], mood and affect assessment [144], diet and physical activity [145], and obesity-specific contexts [146], with additional activity sensor capabilities that serve to augment “message-based” data for such approaches [147].

Individual tethering accordingly supports personalized medicine or public health interventions as allowing for specific messages to be sent to specific individuals. In general, individually-tailored messages in public health interventions are more likely (when properly designed) to effect behavior change than generic messages [148]. Although the particular definition of “tailoring” in public health and health care has varied widely [149,150], our interpretation simply addresses the fundamental characteristic of what the technology can deliver—connectivity tethered to an individual. How any form of tailoring will be used (and its likely impact will be) exploiting that characteristic depends on the quality of the design in the context of an intervention. For example, linking an ecological momentary assessment method with an intervention treatment to support individually-tailored SMS connectivity [151], but use of other forms of tailoring exploiting the tethered nature of mobile phones are being developed, such as with human immunodeficiency virus adherence [152], but is a growing component of many SMS intervention designs [153]. Again, the tethered technology affords various types of tailoring, but the impact of any tailoring depends on the quality of the design and its implementation. Of course, the 1:1 mapping also allows for large amounts of (and of potentially high fidelity) *patient-generated health data*, aggregated for location and time-dependent analyses at a group level, such as disease surveillance or clinical practice statistics [154,155].

A question regarding tethering involves not only the generation of individual’s data but also one of access to individual’s EHR. A survey by Accenture [156] demonstrates how both physicians’ and health consumers’ attitudes and behaviors regarding digital technology and health have changed in 2 years, for example, in patient’s actual access their electronic records (2014: 27%, 2016: 45%), in what data they have access to in their EHR (2014: 39%, 2016: 65%), and in the use of health apps (2014: 16%, 2016: 33%). However, the same survey revealed that 92% of patients believed that they should have full access to their EHR, whereas only 18% of physicians held that same belief. This is one example of gaps that may exist between 2 of the key stakeholders with respect to use of this technology: patients and their health care providers.

### Characteristic Interactions and Their Consequences

Of course, the world is often more complex and dynamic than we either describe or anticipate. Collectively, interactions and secondary impacts of these characteristics are beginning to emerge. For example, the problem of individuals’ “access to access” (ie, availability of technology that enables access to health services) is evaporating. As the penetration of smartphone into all populations continues, it affords an important vehicle for recruitment of under-represented groups in clinical trials and other health-related research [157]. But as technology forms become more effective and efficient, so do mHealth offerings, and this usually results in differential access (eg, via cost) to the “best” mHealth solutions [158].

Mobile phone–based apps allow the localization of data acquisition, processing, and presentation or response options to specific individuals, situations, and context. Access to the Internet or World Wide Web increases the amount and forms of data gathered and transmitted to and from individuals and groups, over time and across place. Consequently, mHealth for health care has generated many variations of technological use, ranging from the ability to generate tailored messages to individuals or to groups of individuals with similar needs via the Web [159] or via text messages [160], to engaging technological combinations such as those being designed in broad, social media systems [161] affording “peer-to-peer” health care [162] or to realizing new health surveillance capabilities through extant technology and relevant populations, such as “participatory epidemiology” forms of engagement and rapid communication [163-165].

The rise of inter-connectivity of local health-rated devices will not only increase the potential of mHealth but also increase the complexity of its design. The nature of “wearable” health devices are currently worn either on the wrist (55%) or the chest (27%) or the purse or pocket or shoe (17%), but are also appearing on the arm (8%), head (7%) clothing (6%) leg or ear (5%), ankle (3%), necklace (3%), or finger (1%) [109] and are highly dependent on sensors [166]. The global wearable health device market between 2015-2020 is projected to achieve revenue growth of $41.3 billion by 2020 [167]. Pennic [168] cites a MarketsandMarkets report projecting the ingestible sensor market to grow to $678 million by 2020. Data captured by these devices can form a personal, integrated health sensor network that could be easily collected, integrated, and analyzed by mobile phone technology.

The rapid development of mHealth technologies has resulted in market interposition wherein traditional providers of health care services have been partially displaced by preferred technological self-treatment [169]. In fact, Price-Waterhouse-Coopers placed “Do-it-yourself healthcare” as their top health industry issue for 2015 [170] and places “care in the palm of your hand” as the third top industry issue for 2016 [171]. However, this may speak less about preference of care and more about ease of access. Given the opportunities for engaging mHealth apps for public health, it is necessary to understand the broad array of interconnection forces at play, now and likely in the future. This is a distinctly different patient-facing functionality than the failed Google Health’s medical records or health data platform [172] offering various forms of disintermediating from “traditional” health care reimbursement, face-to-face models, but it is difficult to predict how the insurance industry or federal or state regulators will respond.

What is clear is that the upcoming Stage 3 requirement of the Meaningful Use incentive program from the Centers for Medicare and Medicaid Services strongly encourages patient engagement (care management, wellness, and patient supplied data) outside of a clinic using mobile health apps, coordination of care, and patient access to health information via mobile platforms [173]. Recall that Apple’s HealthKit integrates with the Epic System’s EHR system. Consider that one of the primary reasons for HealthKit’s growing adoption in health systems is the fundamental simplicity of connecting to institutional EHR systems [174], and the top EHR vendor meeting the Meaningful Use requirement is Epic [175]. Note also that Duke Medicine recently succeeded in being the first Epic-based health system to implement the Fast Healthcare Interoperability Resources standard with Apple’s HealthKit [176], a standard that also allows Epic Systems EHR data to interoperate with IBM’s Watson Health analytics [177]. Overall, the adoption of even basic EHR by the key gatekeepers, the physicians, is occluded by the encountered difficulties of data exchange, whether it involves interoperability standards or strategic moves by vendors to gain competitive advantage [178]. Nevertheless, specific inroads work to demonstrate ways of overcoming such obstacles for specific health objectives on mobile apps [179-181].

## Conclusion

mHealth is an ill-defined, but growing component of all aspects of health (prevention, diagnosis, treatment, research) around the world. We focused on 4 core characteristics of the foundational platform, the mobile smartphone, that will likely remain as important to understand to help design and implement more effective and efficient mHealth solutions. More importantly, as each characteristic has surprising depth, complexity, and relation to the other characteristics, it is essential to discern how these work together in any particular mHealth context. Each type of characteristic embodies both features and constraints.

This is the fastest spreading communication technology. The first core characteristic—penetration into populations—is possible because mobile telephony is becoming ubiquitous, and this has significant implications for reach into populations, although not homogenously in many cases. Therefore, is necessary to know the technological platform most adopted and how it is used by the target population or subgroup.

Apps are powerful but issues of use are complicated. The second core characteristic—the availability of app *—* affords potentially sophisticated function (and computation) available to the device, depending on the type of mobile phone. In addition, the growth of short-range device connectivity allows ancillary devices to be connected to the mobile phone, enlarging the set of technological options in communication, monitoring, and delivery of health solutions. In addition, both the sophistication of sensors on, or connected to, a mobile phone raises the question of whether it is (they are) a medical device (in a legal sense) and how HIPAA rules guide the storage and use of that data. Given the immense growth in apps, competition can be intense, but opportunities for innovation are practically unbounded.

Connectivity matters. The third characteristic—wireless broadband—is the most rapidly growing technological platform for Internet access, so consideration of mHealth contexts with Internet connectivity, even intermittent connectivity, affords a substantially different pathway for expected data rates to and from devices, influencing the formation and functionality of mHealth apps and their target groups. However, as lower income groups increase their (often sole) reliance on mobile broadband for Internet connectivity, data charges can escalate rapidly. Costs to the client are still a part of the use equation.

Almost everybody has a mobile phone. The fourth characteristic—that mobile phones are generally tethered to individuals—affords direct access to individuals allowing deep penetration to the individual level, with apps being tailored to individuals and having the ability to adapt to individual behaviors and physiological data, captured and communicated distally (if necessary) in real time. The primary interpretation of the 4 characteristics is that they serve as both opportunities and constraints. However, to exploit them as either, it is essential to understand what they are, and how they relate to one’s *specific* mHealth context. Regardless, they are the enduring characteristics common to all mHealth mobile phone apps.

Recently Gordon Moore (the architect of Moore’s Law) stated, *We’ve just seen the beginning of what computers are going to do for us* [182]. The ubiquity of the devices, their computational ability, and their ability to capture and rapidly transmit complex data, whether the data comes from “medical devices (Food and Drug Administration approved) or not, the rate and amount of data captured lends itself to large-scale analytics affording new, broader capabilities” [183] which, of course, can be used for “good or evil” [184]. Along with “Big Data” often comes “Big Noise,” so the merging of technology and health contexts will allow us the opportunity to move from collecting *data*, which has a cost (eg, acquisition, storage, transfer, security, integrity), to efficiently extracting *information*, which as a value by guiding decisions that lead to health improvements in individuals, groups, and societies. The key is achieving the economically-viable potential to turn acquired data into information that can be applied to the betterment of health, which necessitates not only well-designed interventions but also well-designed evaluations of those interventions [185]. Yet, there is likely another level where health care, and public health, use that information to inform policies that are enabled, and not blocked, by institutional mechanisms. Still the fundamental mantra remains: mHealth is not about technology; it is about how technology is appropriately used in the context of achieving specific health goals [186,187]. Nevertheless, future discussions must include additional considerations of how to weave the use and usability of such technology into the foundational designs of interventions, and not simply a delivery platform afterthought, as technology is not neutral. Translating evidence-based interventions for one mode in which it was designed (eg, workshops) cannot be adopted for another mode without determining the impact on the integrity of the original logic [188,189].

Mobile phone and communication technologies afford us characteristics with remarkable capabilities and potential. And these are rapidly, unpredictably, and even disruptively, changing and changing health care and public health. Are we ready?

## Abbreviations

ECG: electrocardiogram
EHR: electronic health record
HIPAA: Health Insurance Portability and Accountability Act
ICT: Information and Communications Technologies
ITU: International Telecommunications Union
OS: operating system
SD: secure digital
SDK: systems development kit
SMS: short messaging service
